# Normal Values in Esophageal High-Resolution Manometry Performed Using 36-Channel Water-Perfused Catheter or Solid-State Catheter

**DOI:** 10.5152/tjg.2025.24582

**Published:** 2025-03-25

**Authors:** Serhat Bor, Anahita Sadeghı, Sezgi Kıpcak, Ali Senkaya

**Affiliations:** 1 Division of Gastroenterology, Ege University Medical School, İzmir, Türkiye; 2 Digestive Research Institute, Tehran University of Medical Sciences, Tehran, Iran; 3 Department of Medical Biology, Ege University Medical School, İzmir, Türkiye; 4 Department of Gastroenterology, Ministry of Health İzmir City Hospital, İzmir, Türkiye

**Keywords:** Esophageal motility disorders, manometry, water-perfused high-resolution manometry, solid-state high-resolution manometry

## Abstract

**Background/Aims:**

Manometric measurements are crucial for diagnosing esophageal motility disorders. High-resolution manometry (HRM) studies mainly use 2 catheter systems: solid state (SS) and water perfused (WP), each with distinct advantages. This study aimed to establish normal values for esophageal manometry using both 36-channel WP and SS catheters in healthy volunteers.

**Materials and Methods:**

This study, conducted between January 2017 and September 2018, included 44 healthy volunteers with no upper gastrointestinal symptoms or history of gastrointestinal surgery (except inguinal hernia repair or appendectomy). Participants gave written informed consent, abstained from medications and alcohol, and underwent normal endoscopy. They then had 2 consecutive esophageal manometry sessions, 1 day apart, using a 36-channel SS-HRM catheter and a 36-channel WP-HRM catheter. All tracings were analyzed using the Chicago classification version 3.0.

**Results:**

Four participants were excluded due to gastroesophageal reflux disease (GERD). Of the remaining 40 (age 37.4 ± 7.6, 62.5% male), all underwent WP-HRM, and 34 underwent SS-HRM. In SS-HRM, 74 of 386 swallows were <450 mm Hg·s·cm; in WP-HRM, 151 of 441 swallows were <<450 mm Hg·s·cm. Thus, 4 of 34 volunteers (11.8%) in SS-HRM and 12 of 40 (30%) in WP-HRM had ≥50% swallows with DCI <450 mm Hg·s·cm. Median IRP4 was 17 (7-27) mm Hg in SS-HRM vs. 6 (0-18) mm Hg in WP-HRM. The 5th-95th percentile DCI was 183-2962 mm Hg·s·cm in SS-HRM vs. 65.5-1711.5 mm Hg·s·cm in WP-HRM.

**Conclusion:**

This study compares normal values and differences in WP-HRM and SS-HRM among healthy Turkish volunteers, demonstrating differing diagnostic criteria and providing valuable reference data for future studies.

**Main Points**

- This study establishes normal esophageal manometry values for both 36-channel WP and SS catheters in healthy Turkish volunteers.
- The study demonstrates significant differences in diagnostic thresholds for integrated relaxation pressure (IRP) and distal contractile integral (DCI) between WP-HRM and SS-HRM.
- Water-perfused high-resolution manometry showed a higher percentage of ineffective swallows compared to SS-HRM, potentially leading to overdiagnosis of ineffective esophageal motility disorders.
- The findings highlight the importance of adjusting diagnostic criteria based on catheter type to avoid misdiagnosis in clinical practice.
- This reference data provides a valuable basis for future research and clinical applications involving esophageal HRM in different populations.

## Introduction

Functional esophageal disorders are common in the general population and cause significant morbidity. Manometric measurements, which are among the most important methods for diagnosing esophageal motility disorders, have become more popular and relevant in recent years with the development of new technologies. Clinically, esophageal manometry is indicated to evaluate dysphagia, odynophagia, achalasia, hypercontractile esophageal motility disorders, non-cardiac chest pain, and gastroesophageal reflux disease (GERD), as well as to assess patients before anti-reflux surgery.^1^^-^^4^ High-resolution manometry (HRM) is a newer method that is faster and easier to apply than conventional manometry.^1^ High-resolution manometry studies mainly use 2 types of catheter systems: solid state (SS) and water perfused (WP), which have different advantages and disadvantages. Water-perfused catheters are more difficult to set up and use, but they are also more comfortable, cheaper, flexible, and thinner than SS catheters on the other hand, SS catheters have advantages over WP catheters, such as faster response, higher resolution, and easier handling therefore, it is considered the gold standard for esophageal HRM.^5^

Water-perfused high-resolution manometry system is a low-cost option for esophageal manometry, especially in developing or under-developed countries. It uses a disposable silicone catheter with multiple water-filled channels that are connected to external transducers by a pneumatic pump. This system enhances patient safety and reduces the risk of infection. However, WP catheters have some drawbacks, such as being prone to channel obstruction, requiring more time for installation and calibration, and needing skilled staff for maintenance. Water-perfused catheters can be sterilized by autoclave.^5^

The Chicago Classification is the standard protocol for evaluating esophageal motility with HRM.^6^^-8^ It defines the normal values for various metrics based on data from Western populations in supine posture using SS catheters.^9^

However, these values may not be applicable to other populations or catheter types. There are limited studies on the normal values obtained by WP catheters in healthy volunteers as well as SS catheters.^10^ ^11^

Moreover, most of these studies only screened for symptoms and did not exclude other potential causes of esophageal dysfunction, such as silent gastroesophageal reflux disorder, by using upper gastrointestinal endoscopy and/or 24-hour intra-esophageal pH-impedance monitoring. Therefore, this study aimed to determine the normal values of esophageal manometry performed by both 36-channel WP and SS catheters in the same cohort of healthy volunteers in addition to upper gastrointestinal endoscopy and 24-hour pH-impedance monitoring. Therefore, this study aimed to determine the normal values of esophageal manometry performed by both 36-channel WP and SS catheters in the same cohort of healthy volunteers

## Materials and Methods

This study, conducted between January 2017 and September 2018, included 44 healthy volunteers with no upper gastrointestinal symptoms or history of gastrointestinal surgery (except inguinal hernia repair or appendectomy). The study was approved by the Ege University Ethical Committee (Ethics approval number: 17-4.1/8, date: 08.05.2017). Forty-four healthy volunteers without any upper gastrointestinal symptoms or history of gastrointestinal surgery (except inguinal hernia repair or appendectomy) were recruited and gave written informed consent. They were instructed to stop any medication that could affect upper gastrointestinal motility at least 1 week before the study and to avoid alcohol consumption 1 day before and on the day of the study.

All participants underwent upper gastrointestinal endoscopy to rule out any structural or mucosal abnormalities. Those who had normal endoscopy results were then scheduled for 2 consecutive esophageal manometry sessions with 1 day interval, using a 36-channel unidirectional SS-HRM catheter [Medical Measurement Systems (MMS)-Unisensor—HRM K 123659-00-1002 D] and a 36-channel multidirectional WP HRM catheter (MMS—HRWM CE4-1194), respectively. Before the procedures with WP catheters, systems were carefully examined and amount of water flow was measured. Both catheters were calibrated and inserted through the nose after applying local anesthetic spray, while the participants were in supine position. The catheters were positioned such that at least 2 distal channels were in the stomach. The participants were asked to swallow 5 mL of water every 20-30 seconds until 10 complete swallows were recorded. The data were acquired and stored on a personal computer using a sample frequency of 25 Hz (MMSs, Enschede, The Netherlands). All manometric tracings were manually analyzed using the Chicago classification version 3.0 (version of software 9.3).

The following parameters were measured in HRM recordings: lengths of upper esophageal sphincter (UES), lower esophageal sphincter (LES) and esophageal body; resting pressures of UES and LES; integrated relaxation pressure (IRP); distal contractile integral (DCI); distal contraction latency (DL); peristaltic break; percentage of ineffective peristaltic waves; and intragastric pressure. Integrated relaxation pressure was defined as the minimal median pressure during a 4-second relaxation period and expressed as mm Hg. Distal contractile integral quantified the length, vigor, and persistence of post-deglutitive pressurization in the distal esophageal segment and expressed as mm Hg·s·cm.^6^^,7^ If distal esophageal contraction did not occur following a successful swallow, this contraction was also included in the analysis. The normative cut-off values for each parameter were derived from the literature as follows: IRP (less than 95th percentile), DL (minimum value), DCI (5th-95th percentile: normal; >95th percentile: jackhammer esophagus; 5th-10th percentile: weak; and <5th percentile: failed), and peristaltic break (≤5 cm and >5 cm by segment: proximal or distal). After the second manometry session, all participants underwent a 24-hour esophageal multichannel intraluminal impedance and pH (MII-pH) test to exclude any silent gastroesophageal reflux disorder.

### Statistical Analysis

Data were analyzed using the PASW Statistics for Windows, Version 18.0 (SPSS Inc., Chicago, IL, USA). Descriptive statistics were expressed as mean ± standard deviation, median, minimum–maximum, and 5th-95th percentile for numerical variables. The Bland-Altman method was used to assess the agreement between WP-HRM and SS-HRM in 34 volunteers who underwent both HRM.

## Results

Out of the 44 volunteers, 4 were excluded from the study as they had GERD according to the upper gastrointestinal endoscopy or MII-pH results (9.1% of the healthy volunteers). The remaining 40 participants (age 37.4 ± 7.6, 25%-62.5% male) underwent WP-HRM and 34 of them also underwent SS-HRM. The results of WP-HRM and SS-HRM are presented in Table 1.

In SS-HRM, 74 out of 386 swallows were ineffective and in WP-HRM, 151 out of 441 swallows were ineffective. Therefore, in SS-HRM, 4 out of 34 volunteers (11.8%) and in WP-HRM, 12 out of 40 volunteers (30%) had ≥50% swallows with DCI <450 mm Hg·s·cm. Six volunteers were found to have ineffective esophageal motility according to the CCV3.

The HRM metrics for the supine position are presented in Table 1 for 40 healthy volunteers from our study. The median (min-max) IRP4 was 17 (7-27) mm Hg in SS-HRM compared to 6 (0-18) mm Hg in WP-HRM. The 5th-95th percentile of DCI was 183-2962 mm Hg·s·cm in SS-HRM compared to 65.5-1711.5 mm Hg·s·cm in WP-HRM. The DL (mean ± SD) was 7.5 ± 0.9 seconds in SS-HRM compared to 7.2 ± 1.4 seconds in WP-HRM. The LES resting pressure (mean ± SD) was 35.1 ± 13.2 in SS- HRM compared to 18.5 ± 7.4 in WP-HRM.

### Agreement Between Solid-State High-Resolution Manometry and Water-Perfused High-Resolution Manometry

The results of the Bland-Altman plot analysis performed to assess the agreement between the SS-HRM and WP-HRM for the measurements of LES-resting, IRP (5 mL), DCI esophageal length (inspiration), and inefficient peristalsis percentage are presented in Table 2.

The mean bias value was between −7.26 and 42.482 for LES-resting pressure, between 0.47 and 21.81 for IRP, between −534.79 and 1648.02 for DCI, between −1.61 and 2.67 for esophageal length (inspiration), and −47.30 and 65.07 for inefficient peristalsis percentage.

A moderate degree of reliability was found between SS-HRM and WP-HRM for LES-resting pressure, IRP, and DCI measurements. The average intraclass correlation coefficient (ICC) values were 0.128 (95% CI: −0.088 to 0.386) for LES-resting pressure, 0.061 (95% CI: −0.057 to 0.243) for IRP, and 0.445 (95% CI: −0.037 to 0.729) for DCI. A high degree of reliability was found between SS-HRM and WP-HRM for esophageal length (inspiration) measurements with the average ICC value of 0.831 (95% CI: 0.646-0.918). A poor degree of reliability was found between SS-HRM and WP-HRM for inefficient peristalsis percentage measurements. The average ICC value was 0.005 (95% CI: −0.302 to 0.325).

## Discussion

This study compared the normal values of SS and WP catheters in the healthy volunteers of Turkish people—Izmir, Türkiye—in 2 consecutive days, after excluding any gastrointestinal symptoms or disorders by upper gastrointestinal endoscopy and 24-hour MII-pH test. We found that the upper limit of IRP was 26 mmHg for SS-HRM and 15 mm Hg for WP-HRM, suggesting that the normal values for IRP should be adjusted for these catheters. We also found that WP-HRM showed more ineffective peristaltic waves than SS-HRM, which may lead to overdiagnosis of ineffective motility disorder. Moreover, we found that DCI values were lower in WP-HRM than in SS-HRM, which may result in underdiagnosis of hypercontractile disorders, such as jackhammer esophagus or overdiagnosis of ineffective motility disorders such as related with GERD. Esophageal motility studies use devices and catheters from different manufacturers and configurations. The Chicago classification, which is widely used in esophageal motility disorders, defines the normal values for various metrics based on data from a single device and catheter type. The upper limit of IRP is considered to be 15 mm Hg according to the results obtained from another motility system manufacturer, and any higher value is considered as a major pathology indicating achalasia or esophagogastric junction outflow obstruction. The DCI value of <450 mm Hg·s·cm is also considered as a pathological condition. These metrics are important for the diagnosis of major and minor motility disorders of the esophagus. However, different devices and catheters may have different normal values, depending on their technical specifications and calibration methods. Many users may not be aware of these differences and may use the same normal values regardless of the system or the catheter they have been using. This may lead to inaccurate diagnosis and treatment of esophageal motility disorders. Therefore, it is important to establish the normal values for each device and catheter type based on data from healthy volunteers.^12^ This is specifically true for WP motility systems since many normal values have been obtained from SS systems. Head-to head comparative to studies in same patients between those 2 systems are also lacking. In this study, we used the system from MMS/Laborie Company together with the SS-HRM and WP-HRM catheters in 2 different configurations, which are manufactured by Unisensor and widely used. Our study has some advantages over the previous studies on normal values. First, we selected healthy volunteers among asymptomatic subjects as well as normal upper GI endoscopic examination and 24-hour MII-pH test. This detail, which is frequently ignored in the studies, is important as it allows elimination of various pathologies, primarily silent GERD. Indeed, 4 cases in this study were excluded because of GERD. This rate is even lower than the rate of erosive esophagitis detected by upper GI endoscopy in asymptomatic cases, which is about 10% in some studies.^13^ It is known that GERD can affect DCI and LES-resting pressure.^14^ Therefore, attention should be paid to the results of the studies conducted only in asymptomatic volunteers without excluding GERD by objective tests. Second, we measured both WP-HRM and SS-HRM in the same volunteers on 2 consecutive days, which minimized the inter-individual variability and allowed direct comparison between the 2 systems. Third, we measured all relevant parameters for esophageal motility, including esophageal length and ineffective peristalsis percentage, which were not reported in some earlier studies. We also included all swallows in DCI calculation, regardless of their effectiveness, which we think is a correct technique. In this study, particularly the data obtained by WP catheter indicated that 30% (12/40) of the healthy volunteers had ≥50% swallows with DCI <450 mm Hg·s·cm, which would qualify them for ineffective motility disorder according to the Chicago classification. In this study, we found similarities and differences between 36-channel WP catheter and SS catheter in terms of HRM metrics. In line with a previous study using a 4-channel catheter (12), we found that LES pressure was higher in SS-HRM (35.1 ± 13.2 mm Hg) than in WP-HRM (18.5 ± 7.4 mm Hg). We also found that IRP was higher in SS-HRM than in WP-HRM, which is consistent with some studies but not others. The normal values for IRP obtained by various studies using different catheters are shown in Table 3. We found that DCI was lower in WP-HRM than in SS-HRM, but DL was similar in WP-HRM and SS-HRM, which is in accordance with some studies. The normal values of parameters of esophageal HRM reported in various studies using different catheters are shown in Table 3.

Agreement analysis revealed a poor degree of reliability between SS-HRM and WP-HRM for LES-resting pressure, IRP, and DCI and a high degree of reliability for esophageal length (inspiration). However, there was a poor degree of reliability between the 2 systems in terms of inefficient peristalsis percentage. These results suggest that WP-HRM and SS-HRM may not be interchangeable for some measurements and that the normal values should be adjusted for each system. Water-perfused high-resolution manometry catheters and their systems usually belong to a single manufacturer. Even so, the normal values of IRP vary between 2.5 and 23.5 mm Hg^15^ ^16^ and the normal values of LES resting pressure vary from 5 to 54 mm Hg.^15^ ^17^

In all studies, interestingly, the lower limits of DCI are extremely low. Therefore, the diagnostic criteria for ineffective peristalsis and jackhammer esophagus should be re-evaluated for WP-HRM. It should also be noted that normal values of WP and SS catheters are quite different in clinical practice. In some centers, both catheters are used in the same system. In this case, the normal values should be changed according to the catheter type.

This study and similar studies are of critical importance in clinical practice. Unfortunately, some centers only rely on the numbers without taking care of differences related to fit systems or catheters, resulting in wrong diagnoses. Those mistakes might result in dangerous invasive procedures. It is an important responsibility of the industry to provide the normal values for every catheter and system they produce and distribute. Furthermore, meta-analyses of the studies investigating the same catheter type are needed to establish more robust normal values.

The limitations of this study are the use of a single device and catheter type from one manufacturer, which may limit the generalizability of the results to other devices and catheters; the lack of measurement in the upright position, which may affect some HRM metrics; and the lack of provocative tests, which may reveal latent motility disorders.

In conclusion, our study compares the normal values and differences of WP-HRM and SS-HRM in a large and well-selected group of healthy Turkish volunteers. Our study demonstrates that WP-HRM and SS-HRM have different diagnostic values and thresholds for esophageal motility disorders and also provides valuable reference data for future studies that use WP-HRM or SS-HRM in different populations or settings.

## Figures and Tables

**Table 1.** Results of Water-Perfused High-Resolution Manometry and Solid-State High-Resolution Manometry of the Volunteers

	SS-HRM				WP-HRM			
	N	Mean ± SD	Median (min-max)	5th-95th Percentile	N	Mean ± SD	Median (min-max)	5th-95th Percentile
UES parameters								
Resting pressure, mm Hg	34	98.3 ± 52.0	92 (26-225)	32-219	39	100 ± 52.7	96 (25-223)	30-193
IRP 0.2 s, mm Hg	34	1.3 ± 5.3	1 ([−10]-16)	(-8)-11	39	18.0 ± 9.9	16 (1-55)	7-44
IRP 0.8 s, mm Hg	34	28.4 ± 11.6	27.5 (8-55)	9-51	39	28.4 ± 11.0	28 (1-61)	9-53
Length	34	3.0 ± 0.5	2.8 (1.9-4.2)	2-4	39	3.4 ± 0.6	3.3 (2.4-5.6)	2.9-5.0
LES parameters								
Resting pressure, mm Hg	34	35.1 ± 13.2	36 (13-68)	14-64	40	18.5 ± 7.4	17.5 (2-36)	8-31.5
IRP 4s, mm Hg	34	17.7 ± 5.1	17 (7-27)	10-26	40	7.04 ± 4.05	6 (0-18)	1-15.5
Length, cm	34	2.9 ± 0.5	2.9 (1.9-3.8)	2.0-3.7	40	2.9 ± 0.6	3 (1.6-3.9)	2.1-3.9
Esophageal parameters								
DCI, mm Hg·s·cm	34	1297.2 ± 752.6	1080 (36-3390)	183-2962	40	718.1 ± 477.6	648.5 (34-2251)	65.5-1711.5
DL, s	34	7.5 ± 0.9	7.4 (5.8-9.4)	5.9-9.2	39	7.2 ± 1.4	7.2 (1.4-10.1)	5.6-9.5
Length (inspiration), cm	34	22.9 ± 2.0	22.9 (18.4-26.3)	18.8-26.2	40	22.4 ± 1.9	22.1 (18.2-26.8)	18.9-25.3
Length (expiration), cm	34	21.8 ± 2.0	21.7 (17.2-25.2)	17.2-24.6	40	21.5 ± 2.1	21.5 (17.2-28.1)	18.1-24.7
Peristaltic break	34	2.7 ± 2.7	2.2 (0-10.7)	0-10.6	40	3.5 ± 2.9	2.6 (0-11.7)	0-8.4
Ineffective peristalsis percentage	34	19.1 ± 25.5	9 (0-100)	0-100	40	33.8 ± 34.8	25 (0-100)	0-100

DCI, distal contractile integral; DL, distal contraction latency; IRP, integrated relaxation pressure; LES, lower esophageal sphincter; min-max, minimum-maximum; SD, standard deviation; SS, solid-state high-resolution manometry; UES, upper esophageal sphincter; WP, water perfusion high-resolution manometry.

**Table 2.** Agreement between Water-Perfused High-Resolution Manometry and Solid-State High-Resolution Manometry (Bland–Altman Analysis)

	N	SS-HRM	N	WP-HRM	Difference		95% Limits of Agreement			95% CI	
		Median (5th-95th Percentile)		Median (5th-95th Percentile)	Average	SD	Lower Bound	Upper Bound	Intraclass Correlation	Lower Bound	Upper Bound
LES resting pressure, mm Hg	34	36 (14-64)	34	16 (8-32)	17.618	12.685	−7.245	42.480	0.128	−0.088	0.386
IRP 4s, mm Hg	34	17 (10-26)	34	6 (1-15)	11.141	5.443	0.474	21.809	0.061	−0.057	0.243
DCI, mm Hg·s·cm	33	1085 (269-2962)	33	671 (225-2050)	556.618	556.850	−534.787	1648.023	0.445	−0.037	0.729
Esophageal length, cm, (inspiration)	34	22.9 (18.8-26.2)	34	22.2 (18.4-25.5)	0.532	1.093	−1.609	2.674	0.831	0.646	0.918
Ineffective peristalsis, %	34	9 (0-100)	34	25 (0-100)	−12.882	35.569	−82.597	56.833	0.174	−0.140	0.467
DL, sn	33	7.3 (5.9-9.2)	33	7.1 (5.6-9.5)	0.297	1239	−2.132	2.726			

DCI, distal contractile integral; DL, distal latency; IRP, integrated relaxation pressure; LES, lower esophageal sphincter; SS, solid-state high-resolution manometry; UES, upper esophageal sphincter; WP, water-perfusion high-resolution manometry.

**Table 3.** Normal Values of Parameters of Esophageal High-Resolution Manometry Reported in Various Studies

Study	Year	N	DCI	IRP	LES Resting Pressure	Esophageal Length (Inspiration)	Inefficient Peristalsis %	Catheter Type	Company
Solid state									
Sweis et al^18^	2011	23	944.9 (196.8-2433.4)	3.3 [(−0.4)-8.7]	18.9 (5.2-38.4)	–	–	36-channel circumferential sensors 10 mm intervals (ManoScan 360)	Sierra Scientific Instruments
Bogte et al^19^	2013	52	1008.45 (185.65-3407.60)	11.60 (2.59-28.28)	31.00 (8.95-51.40)	–	–	36-channel unidirectional (Unisensor AG)	MMS International
Niebisch et al^20^	2013	68	1485 (420-4236)	9.4 (3-16.7)	22.2 (10.2-43.5)	–	0 (NA-10)	36 channel, each with 12 circumferential pressure sensors.	Sierra Scientific Instruments or Given Imaging
Shi et al^21^	2013	42	1527 (NA-3195)	14.1 (NA-20.5)	–	–	–	32-channel circumferential pressure (DM: 4.2 mm) 5 dual impedance sensors 5 cm apart	Sandhill Scientific Inc.
Zhang et al^22^	2013	24	1508.4 (293.4-3320.0)	7.3 (3.4-12.7)	–	–	–	36-channel circumferential OD: 4.2 mm (Manoscan 360)	Sierra Scientific Instruments
Kessing et al^23^	2014	50	970 (141.6-3674)	7.0 (1.0-18.8)	11.6* (3.0-29.8)	–	–	36-channel circumferential	Given Imaging
Weijenborg et al^11^	2014	50	834 (178-2828)	7.0 (2.0-15.5)	15.0 (3.0-31.2)	–	–	36 channel	Given Imaging
Xiang et al^24^	2017	88	–	9.0 (3.6-17.3)	20.4 (10.8-36.2)	24.5 (21.7-28.0)	–	36 channel (OD: 4.2 mm)	Sierra Scientific Instruments
Hiranyatheb et al^25^	2018	41	1152.05 (241.93-2736.60)	7.2 (1.7-12.8)	23.10 (11.9-23.1)	24.9 (22.0-28.4)	0 (0-20.0)	ManoScan 360TM; 36 circumferential OD: 4.2 mm	Sierra Scientific Instruments
Bor et al (Current study)		34	1085 (269-2962)	17.0 (10-26)	36 (14-64)	22.9 (18.8-26.2)	0.09 (0-100)	36-channel unisensor 1 cm spaced	MMS international
Water perfusion									
Burgos-Santamarí et al^15^	2015	16	841 (285-2820)	9.0 (2-20)	27.5 (5-54)	–	–	–	MMS international
Tseng et al^26^	2018	66	799.0 (99.4-2185.6)	4.8 (0-20.0)	19.0 (8.7-46.5)	21.3 (17.6-25.4)	0 (0-86.5)	–	MMS international
Srinivas et al^27^	2018	53	1398 (72-3276)	6.1 (1.3-13.0)	13.0 (4.4-37.6)	–	–	–	MMS international
Kessing et al^23^	2014	50	1189 (141.6-3674)	8.1 (1-18.8)	23.7 (9.1-54.8)	–	–	36-channel Dentsleeve	MMS international
Bor et al (current study)	2024	34	671 (225-2050)	6.0 (1-15)	16.0 (8-32)	22.2 (18.4-25.5)	0 (0-1)	36-channel unisensor 1 cm spaced	MMS international

Data are presented as median (5th-95th percentile); only the data with * is presented as mean. DCI, distal contractile integral; IRP, integrated relaxation pressure; LES, lower esophageal sphincter; OD, Outer Diameter; MMS, Medical Measurement Systems.

## Data Availability

The datasets generated and/or analyzed during the current study are available from the corresponding author upon reasonable request.

## References

[1] Bredenoord AJ Smout AJPM. High-resolution manometry. Dig Liver Dis. 2008;40(3):174 181. (doi: 10.1016/j.dld.2007.11.006) 18155652

[2] Lazarescu A. New diagnostic techniques for esophageal disorders. Can J Gastroenterol. 2008;22(11):903 908. (doi: 10.1155/2008/671434) 19018334 PMC2661191

[3] Pandolfino JE Roman S. High-resolution manometry: an atlas of esophageal motility disorders and findings of GERD using esophageal pressure topography. Thorac Surg Clin. 2011;21(4):465 475. (doi: 10.1016/j.thorsurg.2011.08.007) 22040629 PMC3582185

[4] Bowers SP. Esophageal motility disorders. Surg Clin North Am. 2015;95(3):467 482. (doi: 10.1016/j.suc.2015.02.003) 25965124

[5] Bredenoord AJ Hebbard GS. Technical aspects of clinical high-resolution manometry studies. Neurogastroenterol Motil. 2012;24(suppl 1):5 10. (doi: 10.1111/j.1365-2982.2011.01830.x) 22248102

[6] Kahrilas PJ Ghosh SK Pandolfino JE. Esophageal motility disorders in terms of pressure topography: the Chicago Classification. J Clin Gastroenterol. 2008;42(5):627 635. (doi: 10.1097/MCG.0b013e31815ea291) 18364587 PMC2895002

[7] Pandolfino JE Ghosh SK Rice J Clarke JO Kwiatek MA Kahrilas PJ. Classifying esophageal motility by pressure topography characteristics: a study of 400 patients and 75 controls. Am J Gastroenterol. 2008;103(1):27 37. (doi: 10.1111/j.1572-0241.2007.01532.x) 17900331

[8] Nikaki K Ooi JLS Sifrim D. Chicago classification of esophageal motility disorders: applications and limits in adults and pediatric patients with esophageal symptoms. Curr Gastroenterol Rep. 2016;18(11):59. (doi: 10.1007/s11894-016-0532-y) 27738966

[9] Fox MR Sweis R Yadlapati R Chicago classification version 4.0(©) technical review: update on standard high-resolution manometry protocol for the assessment of esophageal motility. Neurogastroenterol Motil. 2021;33(4):e14120. (doi: 10.1111/nmo.14120) PMC8268048 33729668

[10] Srinivas M Jain M Bawane P Jayanthi V. Normative values for esophageal motility assessed in the physiological seated position for 16-channel water perfused high-resolution esophageal manometry system and postural variations in healthy volunteers. J Neurogastroenterol Motil. 2020;26(1):61 66. (doi: 10.5056/jnm19082) 31917914 PMC6955184

[11] Weijenborg PW Kessing BF Smout AJPM Bredenoord AJ. Normal values for solid-state esophageal high-resolution manometry in a European population; an overview of all current metrics. Neurogastroenterol Motil. 2014;26(5):654 659. (doi: 10.1111/nmo.12314) 24533917

[12] Rengarajan A Drapekin J Patel A Gyawali CP. Comparison of two high-resolution manometry software systems in evaluating esophageal motor function. Neurogastroenterol Motil. 2016;28(12):1836 1843. (doi: 10.1111/nmo.12887) 27353018 PMC5125838

[13] Takeshita E Sakata Y Hara M Higher frequency of reflux symptoms and acid-related dyspepsia in women than men regardless of endoscopic esophagitis: analysis of 3,505 Japanese subjects undergoing medical health checkups. Digestion. 2016;93(4):266 271. (doi: 10.1159/000445713) 27160990

[14] van Hoeij FB Smout AJ Bredenoord AJ. Predictive value of routine esophageal high-resolution manometry for gastro-esophageal reflux disease. Neurogastroenterol Motil. 2015;27(7):963 970. (doi: 10.1111/nmo.12570) 25930019

[15] Burgos-Santamaría D Marinero A Chavarría-Herbozo CM Pérez-Fernández T López-Salazar TR Santander C. Normal values for water-perfused esophageal high-resolution manometry. Rev Esp Enferm Dig. 2015;107(6):354 358. 26031863

[16] do Carmo GC Jafari J Sifrim D de Oliveira RB. Normal esophageal pressure topography metrics for data derived from the sandhill-Unisensor high-resolution manometry assembly in supine and sitting positions. Neurogastroenterol Motil. 2015;27(2):285 292. (doi: 10.1111/nmo.12501) 25557525

[17] Migliore M Deodato G. Clinical features and oesophageal motility in patients with tight fundoplication. Eur J Cardiothorac Surg. 1999;16(3):266 272. (doi: 10.1016/s1010-7940(99)00254-7) 10554841

[18] Sweis R Anggiansah A Wong T Kaufman E Obrecht S Fox M. Normative values and inter-observer agreement for liquid and solid bolus swallows in upright and supine positions as assessed by esophageal high-resolution manometry. Neurogastroenterol Motil. 2011;23(6):509-e198. (doi: 10.1111/j.1365-2982.2011.01682.x) 21342362

[19] Bogte A Bredenoord AJ Oors J Siersema PD Smout AJPM. Normal values for esophageal high-resolution manometry. Neurogastroenterol Motil. 2013;25(9):762 e579. (doi: 10.1111/nmo.12167) 23803156

[20] Niebisch S Wilshire CL Peters JH. Systematic analysis of esophageal pressure topography in high-resolution manometry of 68 normal volunteers. Dis Esophagus. 2013;26(7):651 660. (doi: 10.1111/dote.12027) 23383676

[21] Shi Y Xiao Y Peng S Lin J Xiong L Chen M. Normative data of high-resolution impedance manometry in the Chinese population. J Gastroenterol Hepatol. 2013;28(10):1611 1615. (doi: 10.1111/jgh.12285) 23730912

[22] Zhang X Xiang X Tu L Xie X Hou X. Esophageal motility in the supine and upright positions for liquid and solid swallows through high-resolution manometry. J Neurogastroenterol Motil. 2013;19(4):467 472. (doi: 10.5056/jnm.2013.19.4.467) 24199006 PMC3816180

[23] Kessing BF Weijenborg PW Smout AJPM Hillenius S Bredenoord AJ. Water-perfused esophageal high-resolution manometry: normal values and validation. Am J Physiol Gastrointest Liver Physiol. 2014;306(6):G491 G495. (doi: 10.1152/ajpgi.00447.2013) 24481604

[24] Xiang XL Wang A Tu L The motility of esophageal sphincters during liquid and solid bolus swallows: a multicenter normative value study of high-resolution manometry in China. Neurogastroenterol Motil. 2017;29(1). (doi: 10.1111/nmo.12914) 27665746

[25] Hiranyatheb P Chakkaphak S Chirnaksorn S Lekhaka P Petsrikun K Somboonpun K. Normal values of high-resolution manometry in supine and upright positions in a Thai population. Dig Dis Sci. 2018;63(1):173 183. (doi: 10.1007/s10620-017-4838-x) 29143195

[26] Tseng PH Wong RKM Wu JF Normative values and factors affecting water-perfused esophageal high-resolution impedance manometry for a Chinese population. Neurogastroenterol Motil. 2018;30(6):e13265. (doi: 10.1111/nmo.13265) 29230939

[27] Srinivas M Jain M Bawane P Jayanthi V. Chicago Classification normative metrics in a healthy Indian cohort for a 16-channel water-perfused high-resolution esophageal manometry system. Neurogastroenterol Motil. 2018;30(10):e13386. (doi: 10.1111/nmo.13386) 29856105

